# Increasing BMI is associated with reduced expression of P-glycoprotein (*ABCB1* gene) in the human brain with a stronger association in African Americans than Caucasians

**DOI:** 10.1038/tpj.2016.74

**Published:** 2016-11-29

**Authors:** J Vendelbo, R H Olesen, J K Lauridsen, J Rungby, J E Kleinman, T M Hyde, A Larsen

**Affiliations:** 1 Department of Biomedicine, Health Aarhus University, Aarhus C-DK, Denmark; 2 Center for Diabetes Research, Department of Clinical Pharmacology, Copenhagen University Hospital Gentofte and Rigshospitalet, Copenhagen, Denmark; 3 The Lieber Institute for Brain Development, Maltz Research Laboratories, John Hopkins Medical Campus, Baltimore, MD, USA; 4 Department of Psychiatry & Behavioral Sciences, Johns Hopkins University School of Medicine, Baltimore, MD, USA

## Abstract

The efflux pump, p-glycoprotein, controls bioavailability and excretion of pharmaceutical compounds. In the blood–brain barrier, p-glycoprotein regulates the delivery of pharmaceutical substances to the brain, influencing efficacy and side effects for some drugs notably antipsychotics. Common side effects to antipsychotics include obesity and metabolic disease. Polymorphisms in the *ABCB1* gene coding for p-glycoprotein are associated with more severe side effects to neuro-pharmaceuticals as well as weight gain, indicating a potential link between p-glycoprotein function and metabolic regulation. Using microarray data analysis from 145 neurologically sound adults, this study investigated the association between body mass index (BMI) and *ABCB1* expression in the frontal cortex. Increasing BMI values were associated with a statistically significantly reduced expression of *ABCB1.* Investigation of DNA methylation patterns in a subgroup of 52 individuals found that the methylation/expression ratios of *ABCB1* were unaffected by increasing BMI values. Interestingly, the effect of BMI on *ABCB1* expression appeared stronger in African Americans than in Caucasians.

## Introduction

The human brain is a closed and highly regulated microenvironment. Intracerebral homeostasis is maintained by the blood–brain barrier (BBB), that is, endothelial cells in the tunica intima of the small blood vessels kept together by tight junctions supported by a underlying layer of astrocytic foot processes.^1^ Situated in the luminal membrane of the endothelial cells, P-glycoprotein (P-gp) plays a key role in the barrier.^2, 3^ Here, P-gp functions as an efflux pump pumping its substrates back to the bloodstream, thereby reducing the entry of potentially harmful substances into the extracellular fluid of the nervous system. Simultaneously, P-gp is an important part of the blood–cerebrospinal fluid barrier regulating the content of the cerebrospinal fluid.^4^ Substrates transported by P-gp include pharmacological agents and toxins^5^ as well as certain endogenous substances including lipids and hormones requiring tight regulation.^6^

In addition to the important role for protection of the brain, P-gp is also found in the gut, liver, testis, kidneys and placenta.^5^ The widespread distribution of P-gp underlines its role as an important protective protein in the body, upholding tightly regulated microenvironments and assisting in the elimination of xenobiotic substances.

From a pharmacological point of view the role of P-gp in the BBB is of interest as it may affect the availability of drugs to the central nervous system, hence influencing both the efficacy as well as the adverse effects of neuro-pharmaceuticals. P-gp can be induced or suppressed by various pharmacological agents, including neuroleptics and antidepressants.^5^ Moreover, there are a number of polymorphisms in the *ABCB1* gene coding for P-gp.^7^ Several of these are associated with lowered expression levels of P-gp^8, 9, 10^ or interpersonal variations in the susceptibility to pharmacological treatment or side effects.^11^ Polymorphisms in the *ABCB1* gene have been associated with the occurrence of antidepressant side effects, including sexual dysfunction (1236TT),^12^ hypotension (3435TT)^13^ and serotonergic side effects, for example, sleeplessness,^14^ whereas other polymorphisms (2677G>T) increase the prevalence of side effects of ADHD treatments including insomnia and nightmares.^15^

P-gp polymorphisms have also been associated with metabolic side effects to antipsychotics such as weight gain and increased fasting glucose levels in female schizophrenic patients.^16, 17^

In addition to constituting a common side effect to antipsychotic treatments with widely used drugs such as risperidone and olanzapine,^11^ obesity has become a major health problem worldwide, currently affecting nearly 1.4 billion people.^18^ Interestingly, Ichihara *et al.*^19^ found that the 2677G>A/T polymorphism in the *ABCB1* gene is associated with increased body mass index (BMI) values in otherwise healthy Japanese individuals. This highlights the importance of understanding the role of P-gp in metabolic regulation and the link between P-gp function and the metabolic side effects of several neuro-pharmacological compounds.

In this study, we investigated the relationship between BMI and P-gp (*ABCB1*) gene expression and methylation in the human frontal cortex. All samples in the study were obtained from subjects not suffering from either a neurological or a psychiatric disease. Data were obtained from the BrainCloud database^20^ providing micro- and methylation array data as well as information on gender, race and BMI at the time of death.

## Materials and methods

### Data

The data used in this study were obtained through the BrainCloud database (http://braincloud.jhmi.edu/) courtesy of the Lieber Institute of Brain Development. This database contains methylation and microarray data on post-mortem dorsolateral prefrontal cortex (corresponding to Broadman's areas number 9 and 46) from non-neurological non-psychiatric individuals 0–78 years of age. All microarray expression data were obtained using the Illumina Oligoset (HEEBO7) chip, processed and expressed as previously described.^20^ In addition, methylation data were available from a subset of the individuals based on analysis with the Infinium HumanMethylation27 BeadChips (Illumina, San Diego, CA, USA) as previously described.^21^ All tissue collection was performed with informed consent obtained from the next of kin. All data were subsequently anonymized in accordance with the rules and regulations of the National Institute of Health (NIH) (using protocol 90-M-0142).

### Demographics

The main demographics of the data samples used (at the time of the analysis) are shown in Tables 1 and 2. In the present study, we selected adult individuals ⩾18 years of age. In addition, we excluded four subjects of Asian descent and six of Hispanic descent as well as four subjects with known diabetes and three subjects who lacked information on BMI status, resulting in a total number of 145 adult samples (85 African Americans and 63 Caucasians) to be analyzed (see Table 1 for details).

The influence of BMI on the methylation/expression ratio was examined using methylation data from 52 individuals (26 Caucasian and 26 African American) aged ⩾18 years, excluding Hispanic and Asian individuals, as well as three individuals with known diabetes and one individual missing BMI data (for details see Table 2).

### Statistics

Gene expression values were obtained through the BrainCloud^20^ database (http://braincloud.jhmi.edu/) and analyzed using a multiple regression model, treating BMI and age as continuous variables and race and sex as factors. The association between BMI and P-gp (*ABCB1*) expression was treated as the main outcome of the multiple regression analyses and the potential confounding effects of age, sex or race on the main outcome were explored. To investigate possible interactions between BMI race, age and gender, an additional multiple regression analysis was performed looking at BMI in combination with each of this variables (age, gender, race) separately. Additionally, an analysis of the direct impact of age on P-gp expression was performed co-varying for race and sex. To further evaluate if race or sex affected P-gp expression, we performed analyses of African Americans versus Caucasians and men versus females. The underlying assumptions for the use of the multiple regression model were examined using Q-Q plots of residuals and residual versus fitted plots as part of all analyses.

The potential effect of aging on P-gp expression during adulthood was evaluated by analyzing the data in three groups, that is, young adults, middle-aged adults and adults over the age of 50 years, that is, 18–35, 35–50 and >50 years of age, performing comparisons between groups as analysis of variance analyses. To evaluate if race or sex affected differences among age groups, an additional analysis of covariance analysis was performed.

The impact of BMI on methylation of the *ABCB1* gene was accessed by performing multiple regression analysis on the methylation/expression ratio using the same approach as previously described for the expression data.

A level of significance of 0.05 was considered statistically significant in all analyses. The R software version 3.0.3 (R Foundation for statistical Computing; http://www.r-project.org/) was used for all calculations.

## Results

### P-gp gene expression is reduced with increasing BMI—indications of a stronger effect in African Americans

**Figure 1:**
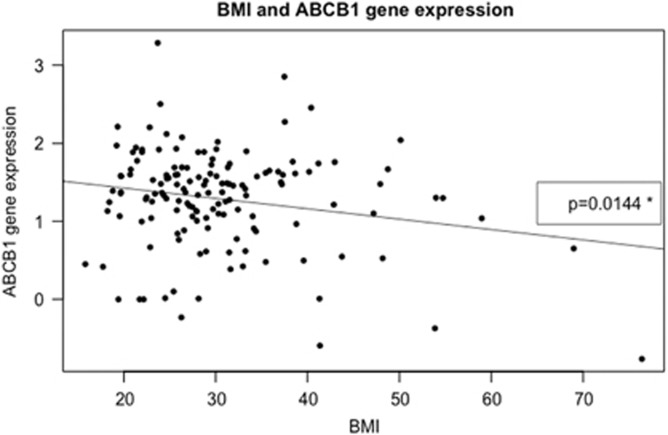
Expression level of P-gp is on the *y*-axis and BMI (kg m^−^^2^) the *x*-axis. All adult individuals with information on BMI and expression levels in the study are included *n=145*; the expression levels are provided in an arbitrary scale. The data are fitted by a linear regression; *y*=1.69179–0.01331 BMI; *P*-value of the slope is 0.0144*.

**Figure 2:**
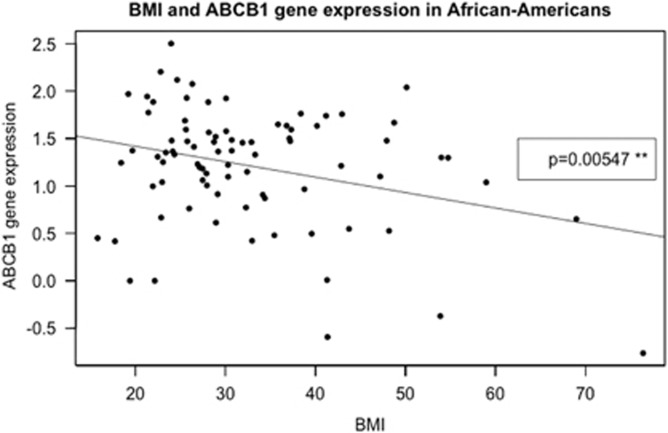
Expression level of P-gp is on the *y*-axis and BMI (kg m^−^^2^) on the *x*-axis. African Americans with information on BMI and expression levels are included (*n=83*). The expression levels are provided on an arbitrary scale. Data are fitted by a linear regression: *y*=1.745109–0.016312 BMI; *P*-value of the slope is 0.00547**. When analyzing the Caucasians of the study alone (*n=62*). The *P*-value of the slopes was 0.2881.

Using a linear regression model we found a statistically significant reduction in P-gp expression with increasing BMI values (*P*=0.014) (see Figure 1). Adjusting for age, race and gender had no significant influence on the association between BMI and P-gp expression. The association between P-gp expression and BMI values was stronger in African Americans when looking separately at African Americans (*P*=0.0055) and Caucasians (*P*=0.2881) (see Figure 2). Accordingly, if analyzing possible interactions BMI–race within a multiple regression model a significant impact (*P*=0.041) of BMI–African-American race was seen. No interactions were seen between gender or age and BMI using this approach.

### P-gp expression is unaffected by age

**Figure 3:**
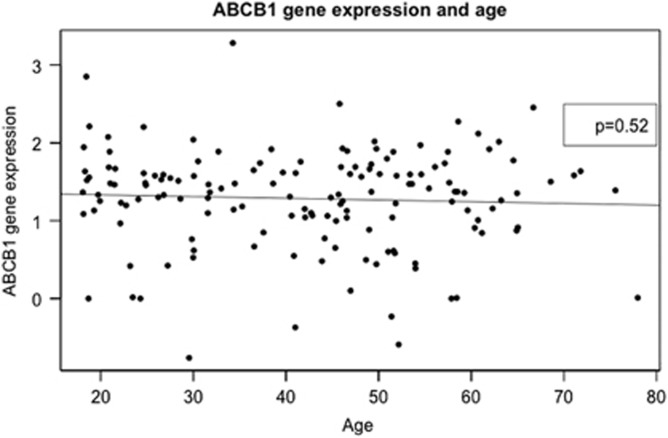
Expression level of P-gp is on the *y*-axis and age on the *x*-axis. All adult individuals with information on age and expression levels in the study are included *n=*52*.*

Looking at age as a continuous variable, no effect on P-gp expression was seen (Figure 3), nor did analysis of variance analysis find any difference of the effect of increasing age when comparing young adults midlife adults and older adults, that is, the three age groups <35 years, 35–50 years and >50 years. Additional analysis of covariance analyses confirmed that differences in sex and race did not affect this result.

### No significant effect of BMI or age on the methylation/expression ratio

**Figure 4:**
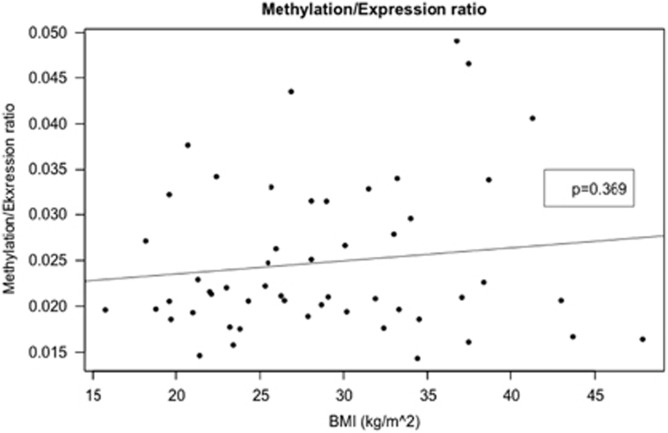
Methylation/expression ratio of P-gp (*ABCB1)* is on the *y*-axis and BMI on the *x*-axis *n*=145. The expression levels are provided in an arbitrary scale. The data are fitted by a linear regression; *y*=1.40–0.0026 Age; *P*-value of the slope is 0.458.

When analyzing the data sets containing information about the methylation/expression ratios, no association was found between BMI or age and the methylation/expression ratio. The data were fitted using linear regression to *y*=1.40–0.0026 Age; *P*-value of the slope is 0.458 (Figure 4).

## Discussion

P-gp is pivotal for the regulation of the central nervous system drug metabolism affecting both the treatment outcome and side effects of pharmacological compounds destined for crossing the BBB. In the present study, we found a significant inverse correlation between BMI values and gene expression of P-gp in otherwise neurologically sound individuals.

Since the major role of P-gp appears to be in regulating substance efflux from the central nervous system,^1^ low expression levels of P-gp may render affected subjects more sensitive to neuro-pharmaceuticals and other substrates of the protein. A number of drugs including risperidone, quetiapine and antidepressants such as citalopram are known P-gp substrates.^22^ As the occurrence of adverse effects is often dose-dependent and drugs are prescribed according to weight, individuals with high BMI might thus be at a higher risk for experiencing adverse effects.

This observation is supported by Sawamoto *et al.*^23^ describing that when treated with Tacrolimus, a known substrate of P-gp, overweight and obese patients needed a significant lower dose to obtain the same blood concentrations as patients with normal BMI. This indicates that the effect of BMI on P-gp expression is not confined to P-gp in the BBB compartment, but may be a universal finding also affecting P-gp expression in other organs systems such as liver, gut, kidney and testis.

The underlying mechanism linking BMI to P-gp expression is unknown. There are several known associations between polymorphisms in the P-gp (*ABCB1*) gene and increasing occurrence of adverse effects to various pharmaceutical compounds, for example, olanzapine,^17, 24^ selective serotonin reuptake inhibitors^12, 14^ and nortriptyline.^13^ Furthermore, a Japanese population study supports that such polymorphisms may be directly linked to the metabolic disturbances and obesity.^19^ Reduced mRNA expression of the *ABCB1* gene might be a direct consequence of circulating pro-inflammatory cytokines in obese individuals. For example, *Il-6* exposure *in vivo* or *in vitro* reduces P-gp gene and protein expression in rat hepatocytes and has a negative effect on P-gp activity.^24, 25^ Moreover, studies on BBB in guinea pigs have shown reduced P-gp mRNA expression in response to both tumor necrosis factor-α, interleukin-1β and interleukin-6 exposure.^26^ Interestingly, this response was seen late in fetal life and postnatally but not in early fetal life. In contrast, studies on the human hCMEC/D3 cell line, a BBB model, revealed only a moderate inverse effect of interleukin-6 on P-gp mRNA expression whereas tumor necrosis factor-α significantly increased P-gp expression.^27^

In rodents, there is a report of a relationship between BMI and P-gp; here the expression levels of P-gp in the small intestine were significantly reduced in obese rats compared with lean rats.^23^ This indicates that P-gp response to certain metabolites generated in obesity might be a causative factor linking high BMI to reduced P-gp expression. Among others, the expression of P-gp in cell membrane microdomains is influenced by several lipids including cholesterol and sphingolipids.^6^ Therefore, an altered lipid profile may affect the expression of P-gp.

If loss of P-gp function leads to increased body weight and P-gp expression is reduced by increasing BMI, a positive feedback mechanism might be created leading to further weight gain. Recently it has also been hypothesized that P-glycoprotein regulates fat metabolism. Foucaud-Vignault and co-workers found that P-gp-deficient mice (mdr1ab^−/−^) had a significantly higher weight gain compared with wild type. These animals also developed hepatic steatosis, when fed the same diet and with the same daily food intake as their wild-type littermates. At the gene expression level, P-gp-deficient mice displayed signs of irregularities in the lipid homeostasis, including an increased *de novo* synthesis of lipids.^28^ Supporting P-gp’s role in lipid regulation, the P-gp genotype 3435TT has been linked to increased levels of ApoA1, that is, the apolipoprotein in high-density lipoprotein particles in healthy individuals.^29^

The role of epigenetic regulation of the *ABCB1* gene has received some attention with regard to the important role of P-gp in the development of resistance to chemotherapy. DNA methylation is an active mechanism in the regulation of *ABCB1* expression.^30^ Epigenetic mechanisms such as DNA methylation could also constitute a possible mechanism by which obesity—as assessed by BMI—could affect P-gp expression.^30^ It is well-known that obesity per se is often related to a variety of potentially stressful responses such as insulin resistance, altered nutritional status and chronic low-grade inflammation.^31, 32^ Moreover, obesity is known to be associated with an increased cancer risk,^32, 33^ which might also reflect deleterious epigenetic alterations. Here we found no evidence for a relationship between P-gp methylation and BMI or age.

As P-gp gene expression can be influenced by the intake of pharmaceutical compounds, one could speculate that differences related to BMI could also reflect differences in the intake of substrates affecting P-gp gene expression. Certain antibiotics such as erythromycin, antimycotics and immunosuppressants are known to inhibit P-gp gene expression,^5^ but the use of such substances is not likely to be affected by body weight/BMI values per se. We cannot exclude the possibility that the use of some inhibitory substrates of P-gp, for example, cardiac drugs like digoxin and verapamil, is unevenly distributed between overweight and normal weight individuals. P-gp also transports a variety of antihypertensives, of which some, like arvedilol, suppress *ABCB1* expression whereas other, like reserpine and nicardipine, induce gene expression. Various 3-hydroxy-3-methyl-glutaryl-coenzyme A reductase inhibitors (statins) are used in the treatment of hypercholesterolemia and at least one statin, Atorvastatin, has been shown to both induce and suppress *ABCB1* (ref. 5) even though others have found that statin treatment and cholesterol reduction do not affect P-gp regulation.^34, 35, 36^

In this data set, the association between BMI and P-gp appeared to be primarily driven by African-American subjects (see Figures 1 and 2). It should however be emphasized that that there was a significant difference between BMI in the two groups—African Americans having a higher BMI than Caucasians (Table 1). Nevertheless, there could be a race difference in the expression of P-gp affecting the association between P-gp and BMI as also indicated in the interaction seen between African-Amercian race and BMI. Earlier studies have shown that the frequency of specific genetic variations in the *ABCB1* gene varies between races.^5^ Ethnic differences are seen in the occurrence of metabolic diseases.^37^ Interestingly, a meta-analysis of BMI and different ethnic groups found small but significant differences in the use of BMI to predict total body fat when comparing ethnic groups with a prediction model made from Caucasian data. Compared with Caucasians with identical BMI values, African Americans had less body fat whereas Indonesian, Ethiopian and Thais had higher body fat. African Americans have a BMI of 1.3 kg m^−^^2^ higher than Caucasians with the same amount of body fat.^37^ If similar racial differences in BMI/body fat relations were present in our data set, this would strengthen the assumption that the P-gp expression reflects genetic and/or epigenetic differences between African Americans and Caucasians. We cannot exclude that psychosocial or socio-economic factors might also contribute to the differences between races reported here. However, the fact that all subjects in the study originated from a relatively small, urbanized area, is likely to limit differences among the populations. Moreover, both racial groups have a similar age profile.

In both the African American and the Caucasian group, there was a slightly uneven gender distribution—that is, more male than female subjects, especially in the Caucasian group. BMI average was similar between the sexes and no gender-based differences were seen in our analyses.

At present, the role of obesity in the development of dementia is under scrutiny. Although recently challenged by the large-scale study of Qizilbash *et al.*,^38^ several studies support that obesity in midlife increases the risk of dementia in later life,^39, 40, 41, 42^ Altered A-beta amyloid metabolism is still considered a major factor in the pathology of Alzheimer's disease, thereby potentially linking BBB function and AD development. Interestingly, double-knockout P-gp null mice (mdr1 a/b−−) have increased A-beta amyloid accumulation in the brain, possibly linking reduced P-gp function to AD pathology.^4, 43^ Similar results have been seen using a P-gp blocker to abolish P-gp function in the BBB on APPswe mice, a type of mice that overexpress human amyloid precursor protein, with a mutation that causes an autosomal dominant form of early-onset familial AD.^4, 43^

In conclusion, here we show that increased BMI is associated with a statistically significant reduced expression of P-gp in the prefrontal cortex of American adults free of significant neurological or psychiatric disorders. The effect of BMI on P-gp *(ABCB1)* expression appeared stronger in African Americans than in Caucasians. With the increased prevalence of obesity in modern society, our findings support a potential role of P-gp in personalized medicine in particular with regard to metabolic side effects. Moreover, the association between increasing BMI and P-gp expression in individuals with no neurological or psychiatric disorders support the notion that BBB dysfunction may also relate to obesity per se.

## Figures and Tables

**Table 1:** Demographics for expression data

*Demographics of the study population*	*All* (n=U5)	*AA* (n=83)	*CAUC* (n=62)	P-*value*
*BMI*				
Mean (kg m^−^^2^)	30.44	32.65	27.48	0.000428
Female	31.84			
Male	29.79			0.2829
Underweight (*n*) (BMI<18)	4	3	1	
Normal weight (*n*) (BMI18–25)	39	19	20	
Overweight (*n*) (BMI 25–30)	41	20	21	
Obese (*n*) (BMI>30)	61	41	20	
				
*Age*				
Mean (years)	41.88	40.76	43.38	0.3054
Young adult (*n*) (age18–35)	54	32	22	
Middle aged (*n*) (age 35–30)	43	25	18	
Elderly adults (*n*) (age>50)	48	26	22	
Age >60 years (*n*)	17	9	8	
				
*Sex*				
Female	46	31	15	
Male	99	52	47	

**Table 2:** Demographics for methylation data

*Demographics*	*All (*n*=53)*	*AA (*n*=26)*	*CAUC (*n*=27)*	P*-value*
BMI, mean (kg m^−2^)	28.73	30.60	26.85	0.06816
Age, mean (years)	46.66	45.66	47.63	0.6872
*Sex*				
Female	25	13	12	
Male	28	13	15	

## References

[bib1] Hermann DM, Kilic E, Spudich A, Krämer SD, Wunderli-Allenspach H, Bassetti CL. Role of drug efflux carriers in the healthy and diseased brain. Ann Neurol 2006; 60: 489–498. 17048260 10.1002/ana.21012

[bib2] Scherrmann J-M. Expression and function of multidrug resistance transporters at the blood-brain barriers. Expert Opin Drug Metab Toxicol 2005; 1: 233–246. 16922639 10.1517/17425255.1.2.233

[bib3] Cordon-cardo C, Brien JPO, Casals D, Rittman-grauert L, Biedler JL, Melamed MR et al. Multidrug-resistance (P-glycoprotein). Proc Natl Acad Sci USA 1989; 86: 695–698. 2563168 10.1073/pnas.86.2.695 PMC286540

[bib4] Rao VV, Dahlheimer JL, Bardgett ME, Snyder a Z, Finch Ra, Sartorelli a C et al. Choroid plexus epithelial expression of MDR1 P glycoprotein and multidrug resistance-associated protein contribute to the blood-cerebrospinal-fluid drug-permeability barrier. Proc Natl Acad Sci USA 1999; 96: 3900–3905. 10097135 10.1073/pnas.96.7.3900 PMC22392

[bib5] Marzolini C, Paus E, Buclin T, Kim RB. Polymorphisms in human MDR1 (P-glycoprotein): recent advances and clinical relevance. Clin Pharmacol Ther 2004; 75: 13–33. 14749689 10.1016/j.clpt.2003.09.012

[bib6] Aye ILMH, Singh AT, Keelan JA. Transport of lipids by ABC proteins: interactions and implications for cellular toxicity, viability and function. Chem Biol Interact 2009; 180: 327–339. 19426719 10.1016/j.cbi.2009.04.012

[bib7] Kroetz DL, Pauli-Magnus C, Hodges LM, Huang CC, Kawamoto M, Johns SJ et al. Sequence diversity and haplotype structure in the human ABCB1 (MDR1, multidrug resistance transporter) gene. Pharmacogenetics 2003; 13: 481–494. 12893986 10.1097/00008571-200308000-00006

[bib8] Hoffmeyer S, Burk O, von Richter O, Arnold HP, Brockmöller J, Johne A et al. Functional polymorphisms of the human multidrug-resistance gene: multiple sequence variations and correlation of one allele with P-glycoprotein expression and activity *in vivo*. Proc Natl Acad Sci USA 2000; 97: 3473–3478. 10716719 10.1073/pnas.050585397 PMC16264

[bib9] Mosyagin I, Runge U, Schroeder HW, Dazert E, Vogelgesang S, Siegmund W et al. Association of ABCB1 genetic variants 3435C>T and 2677G>T to ABCB1 mRNA and protein expression in brain tissue from refractory epilepsy patients. Epilepsia 2008; 49: 1555–1561. 18494787 10.1111/j.1528-1167.2008.01661.x

[bib10] Wang D, Johnson AD, Papp AC, Kroetz DL, Sadée W. Multidrug resistance polypeptide 1 (MDR1, ABCB1) variant 3435C>T affects mRNA stability. Pharmacogenet Genomics 2005; 15: 693–704. 16141795

[bib11] Moons T, Roo Mde, Claes S, Dom G. Relationship between P-glycoprotein and second-generation antipsychotics. Pharmacogenomics 2011; 12: 1193–1211. 21843066 10.2217/pgs.11.55

[bib12] Bly MJ, Bishop JR, Thomas KLH, Ellingrod VL. P-glycoprotein (P-GP) polymorphisms and sexual dysfunction in female patients with depression and SSRI-associated sexual side effects. J Sex Marital Ther 2013; 39: 280–288. 23356581 10.1080/0092623X.2011.615896 PMC3807815

[bib13] Roberts RL, Joyce PR, Mulder RT, Begg EJ, Kennedy MA. A common P-glycoprotein polymorphism is associated with nortriptyline-induced postural hypotension in patients treated for major depression. Pharmacogenomics J 2002; 2: 191–196. 12082591 10.1038/sj.tpj.6500099

[bib14] De Klerk OL, Nolte IM, Bet PM, Bosker FJ, Snieder H, den Boer Ja et al. ABCB1 gene variants influence tolerance to selective serotonin reuptake inhibitors in a large sample of Dutch cases with major depressive disorder. Pharmacogenomics J 2013; 13: 349–353. 22641028 10.1038/tpj.2012.16

[bib15] Kim SW, Lee JH, Lee SH, Hong HJ, Lee MG, Yook K-H. ABCB1 c.2677G>T variation is associated with adverse reactions of OROS-methylphenidate in children and adolescents with ADHD. J Clin Psychopharmacol 2013; 33: 491–498. 23771192 10.1097/JCP.0b013e3182905a8d

[bib16] Kuzman MR, Medved V, Bozina N, Hotujac L, Sain I, Bilusic H. The influence of 5-HT(2C) and MDR1 genetic polymorphisms on antipsychotic-induced weight gain in female schizophrenic patients. Psychiatry Res 2008; 160: 308–315. 18718676 10.1016/j.psychres.2007.06.006

[bib17] Kuzman MR, Medved V, Bozina N, Grubišin J, Jovanovic N, Sertic J. Association study of MDR1 and 5-HT2C genetic polymorphisms and antipsychotic-induced metabolic disturbances in female patients with schizophrenia. Pharmacogenomics J 2011; 11: 35–44. 20195292 10.1038/tpj.2010.7

[bib18] Finucane MM, Stevens GA, Cowan MJ, Danaei G, Lin JK, Paciorek CJ et al. National, regional, and global trends in body-mass index since 1980: systematic analysis of health examination surveys and epidemiological studies with 960 country-years and 9·1 million participants. Lancet 2011; 377: 557–567. 21295846 10.1016/S0140-6736(10)62037-5 PMC4472365

[bib19] Ichihara S, Yamada Y, Kato K, Hibino T, Yokoi K, Matsuo H et al. Association of a polymorphism of ABCB1 with obesity in Japanese individuals. Genomics 2008; 91: 512–516. 18442890 10.1016/j.ygeno.2008.03.004

[bib20] Colantuoni C, Lipska BK, Ye T, Hyde TM, Tao R, Leek JT et al. Temporal dynamics and genetic control of transcription in the human prefrontal cortex. Nature 2011; 478: 519–523. 22031444 10.1038/nature10524 PMC3510670

[bib21] Numata S, Ye T, Hyde TM, Guitart-Navarro X, Tao R, Wininger M et al. DNA methylation signatures in development and aging of the human prefrontal cortex. Am J Hum Genet 2012; 90: 260–272. 22305529 10.1016/j.ajhg.2011.12.020 PMC3276664

[bib22] Linnet K, Ejsing TB. A review on the impact of P-glycoprotein on the penetration of drugs into the brain. Focus on psychotropic drugs. Eur Neuropsychopharmacol 2008; 18: 157–169. 17683917 10.1016/j.euroneuro.2007.06.003

[bib23] Sawamoto K, Huong T, Sugimoto N, Mizutani Y, Sai Y, Miyamoto K. Mechanisms of lower maintenance dose of tacrolimus in obese patients. Drug Metab Pharmacokinet 2014; 29: 341–347. 24670405 10.2133/dmpk.dmpk-13-rg-110

[bib24] Sukhai M, Yong A, Pak A, Piquette-Miller M. Decreased expression of P-glycoprotein in interleukin-1beta and interleukin-6 treated rat hepatocytes. Inflamm Res 2001; 50: 362–370. 11506391 10.1007/PL00000257

[bib25] Sukhai M, Yong A, Kalitsky J, Piquette-Miller M. Inflammation and interleukin-6 mediate reductions in the hepatic expression and transcription of the *mdr1a* and *mdr1b* genes. Mol Cell Biol Res Commun 2000; 4: 248–256. 11409920 10.1006/mcbr.2001.0288

[bib26] Iqbal M, Ho HL, Petropoulos S, Moisiadis VG, Gibb W, Matthews SG. Pro-inflammatory cytokine regulation of P-glycoprotein in the developing blood-brain barrier. PLos One 2012; 7: e43002. 22973436 10.1371/journal.pone.0043022 PMC3433182

[bib27] Poller B, Drewe J, Krähenbühl S, Huwyler J, Gutmann H. Regulation of BCRP (ABCG2) and P-glycoprotein (ABCB1) by cytokines in a model of the human blood-brain barrier. Cell Mol Neurobiol 2010; 30: 63–70. 19629677 10.1007/s10571-009-9431-1 PMC11498628

[bib28] Foucaud-Vignault M, Soayfane Z, Ménez C, Bertrand-Michel J, Martin P-GP, Guillou H et al. P-glycoprotein dysfunction contributes to hepatic steatosis and obesity in mice. PLoS One 2011; 6: e23614. 21949682 10.1371/journal.pone.0023614 PMC3174940

[bib29] Jeannesson E, Siest G, Bastien B, Albertini L, Aslanidis C, Schmitz G et al. Association of ABCB1 gene polymorphisms with plasma lipid and apolipoprotein concentrations in the STANISLAS cohort. Clin Chim Acta 2009; 403: 198–202. 19285054 10.1016/j.cca.2009.02.019

[bib30] Mencalha A, Rodrigues E. Accurate monitoring of promoter gene methylation with high-resolution melting polymerase chain reaction using the ABCB1 gene as a model. Genet Mol Res 2013; 12: 714–722. 23546954 10.4238/2013.March.11.20

[bib31] Suliburska J, Cofta S, Kalmus G, Sobieska M, Samborski W, Krejpcio Z et al. The evaluation of selected serum mineral concentrations and their association with insulin resistance in obese adolescents. Eur Rev Med Pharmacol Sci 2013; 17: 2396–2400. 24065235

[bib32] Bianchini F, Kaaks R, Vainio H. Overweight, obesity, and cancer risk. Lancet Oncol 2002; 3: 565–574. 12217794 10.1016/s1470-2045(02)00849-5

[bib33] Calle EE, Rodriguez C, Walker-Thurmod K, Thun MJ. Overweight, obesity, and mortality from cancer in a prospectively studied cohort of U.S. adults. N Engl J Med 2003; 348: 1625–1638. 12711737 10.1056/NEJMoa021423

[bib34] Supic G, Jagodic M, Magic Z. Epigenetics: a new link between nutrition and cancer. Nutr Cancer 2013; 65: 781–792. 23909721 10.1080/01635581.2013.805794

[bib35] Kenneth KW, To, MH, BT. Expression and activity of ABCG2, but not ABCB1 or OATP1B1, are associated with cholesterol levels: evidence from *in vitro* and *in vivo* experiments. Pharmacogenomics 2014; 15: 1091–1104. 25084202 10.2217/pgs.14.58

[bib36] Fryar CD, Hirsch R, Eberhardt MS, Yoon SS, Wright JD. Hypertension, high serum total cholesterol, and diabetes: racial and ethnic prevalence differences in U.S. adults, 1999-2006. NCHS Data Brief, 2008, pp 1–8. 20423605

[bib37] Deurenberg P, Yap M, van Staveren WA. Body mass index and percent body fat: a meta analysis among different ethnic groups. Int J Obes Relat Metab Disord 1998; 22: 1164–1171. 9877251 10.1038/sj.ijo.0800741

[bib38] Qizilbash N, Gregson J, Johnson ME, Pearce N, Douglas I, Wing K et al. BMI and risk of dementia in two million people over two decades: a retrospective cohort. Lancet Diabetes Endocrinol 2015; 3: 431–436. 25866264 10.1016/S2213-8587(15)00033-9

[bib39] Whitmer RA, Gunderson EP, Quesenberry CPJr, Zhou J, Yaffe K. Body mass index in midlife and risk of Alzheimer disease and vascular dementia. Curr Alzheimer Res 2007; 4: 103–109. 17430231 10.2174/156720507780362047

[bib40] Whitmer RA, Gustafson DR, Barret-Connor E, Haan MN, Gunderson EP, Yaffe K. Central obesity and increased risk of dementia more than three decades later. Neurology 2008; 71: 1057–1064. 18367704 10.1212/01.wnl.0000306313.89165.ef

[bib41] Xu WL, Atti AR, Gatz M, Pedersen NL, Johansson B, Fratiglioni L. Midlife overweight and obesity increases late-life dementia risk: a population-based twin study. Neurology 2011; 76: 1568–1574. 21536637 10.1212/WNL.0b013e3182190d09 PMC3100125

[bib42] Anstey KJ, Cherbuin N, Budge M, Young J. Body mass index in midlife and late-life as a risk factor for dementia: a meta-analysis of prospective studies. Obes Rev 2011; 12: e426–e437. 21348917 10.1111/j.1467-789X.2010.00825.x

[bib43] Cirrito JR, Deane R, Fagan AM, Spinner ML, Parasadanian M, Finn MG et al. P-glycoprotien deficiency at the blood-brain barrier increases amyloid-beta deposition in a Alzheimer disease model mouse. J Clin Invest 2005; 115: 3285–3290. 16239972 10.1172/JCI25247 PMC1257538

